# LACE1 interacts with p53 and mediates its mitochondrial translocation and apoptosis

**DOI:** 10.18632/oncotarget.9959

**Published:** 2016-06-13

**Authors:** Jana Cesnekova, Jana Spacilova, Hana Hansikova, Josef Houstek, Jiri Zeman, Lukas Stiburek

**Affiliations:** ^1^ Department of Pediatrics and Adolescent Medicine, First Faculty of Medicine, Charles University in Prague and General University Hospital in Prague, Prague, Czech Republic; ^2^ Institute of Physiology, Academy of Sciences of the Czech Republic, Prague, Czech Republic

**Keywords:** p53, LACE1, mitochondria, apoptosis, translocation

## Abstract

p53 is a major cellular tumor suppressor that in addition to its nuclear, transcription-dependent activity is also known to function extranuclearly. Cellular stressors such as reactive oxygen species can promote translocation of p53 into mitochondria where it acts to protect mitochondrial genome or trigger cell death via transcription-independent manner. Here we report that the mammalian homologue of yeast mitochondrial Afg1 ATPase (LACE1) promotes translocation of p53 into mitochondria. We further show that LACE1 exhibits significant pro-apoptotic activity, which is dependent on p53, and that the protein is required for normal mitochondrial respiratory function. LACE1 physically interacts with p53 and is necessary for mitomycin c-induced translocation of p53 into mitochondria. Conversely, increased expression of LACE1 partitions p53 to mitochondria, causes reduction in nuclear p53 content and induces apoptosis. Thus, LACE1 mediates mitochondrial translocation of p53 and its transcription-independent apoptosis.

## INTRODUCTION

Tumor suppressor p53 represents a central molecule of a complex cellular stress signaling pathway [1, 2]. When activated, p53 mediates a wide range of cellular outcomes that might include cell cycle arrest, DNA repair, senescence, and programmed cell death [1]. Under normal conditions, p53 is maintained at low levels by continuous ubiquitination and subsequent proteasomal degradation. Upon stimulation by various stress signals, such as DNA damage, hypoxia, and activated oncogenes, p53 is stabilized and accumulates in the cell inducing cell cycle arrest and/or triggers programmed cell death [1, 2]. p53 can mediate apoptosis in a transcription-dependent and transcription-independent manner [3–5]. Within the nucleus, p53 induces transcription-dependent apoptosis by the transactivation of numerous target genes including NOXA, BAX and PUMA. In contrast to well-studied transcription-dependent signaling, the transcription-independent apoptotic pathway of p53 has only recently been discovered [5–7]. Mitochondrial association and translocation of p53 and molecular interactions between p53 and Bcl-2 family proteins in the mitochondria are known to play an essential role in this process [4, 8]. Although primarily known for its fundamental role in maintenance of nuclear genome, p53 also functions in maintenance of mitochondrial DNA (mtDNA), interacting with mitochondrial genome, polymerase-γ and mitochondrial transcription factor A [9–11]. Despite recent advances in characterization of the various cellular roles played by p53, important mechanisms that allow partitioning of its subcellular activity are only poorly understood.

LACE1 is a mammalian homologue of yeast Afg1 (ATPase family gene 1) mitochondrial ATPase, a member of SEC18-NSF, PAS1, CDC48-VCP, TBP family of ATPases [12]. It is an evolutionary well conserved protein that contains an ATP/GTP binding P-loop (Walker A motif) [13, 14]. Investigation of mouse tissues showed that LACE1 expression is markedly upregulated in heart, kidney and lactating vs. inactive breast tissues [14]. LACE1 gene region was found to be downregulated in natural killer (NK) cell neoplasms [15], and an association between bipolar disorder and two SNPs in LACE1 was found [16]. Here we have examined cellular function of LACE1 protein and found that it interacts with p53 tumor suppressor and mediates its mitochondrial translocation and apoptosis.

## RESULTS

### LACE1 is a mitochondrial integral membrane protein

**Figure 1:**
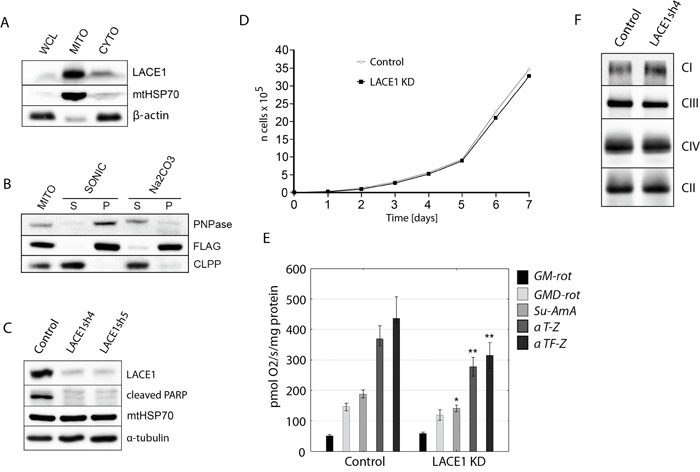
LACE1 is a mitochondrial integral membrane protein whose knockdown by shRNA leads to reduced PARP cleavage and diminished activity of respiratory chain complexes II-IV **A.** LACE1 is a mitochondrial protein. Whole cell lysate (WCL), mitochondrial (MITO) and cytosolic (CYTO) fractions were prepared from HEK293 cells and immunoblotted with antibody to LACE1 (Abcam, UK), to mitochondrial matrix heat shock protein mtHsp70 (Lonza, Switzerland) and to β-actin. **B.** LACE1-FLAG expressed in HEK293 cells is a mitochondrial integral membrane protein. Wild-type HEK293 cells were transiently transfected with a C-FLAG-fusion human LACE1 (NM_145315) ORF construct (OriGene). Submitochondrial fractions were prepared from isolated mitochondria using either sonic disruption (SONIC) or extraction with 100 mM sodium carbonate (pH 11.5). The resulting supernatant (S) and pellet (P) fractions and untreated mitochondria (MITO) were immunoblotted with M2 monoclonal antibody to FLAG epitope (Sigma Aldrich, Germany), and with antibodies to intermembrane space protein PNPase and matrix localized ClpP (Abcam, UK). **C.** Stable knockdown (KD) of LACE1 by shRNA leads to markedly diminished PARP cleavage. Whole-cell lysates (~20 μg of protein) from two different stable shRNA knockdown cell lines (denoted LACE1sh4 and sh5) were immunoblotted with antibodies to LACE1, Cl-PARP, mtHSP70 and α-tubulin. The control cell line was prepared by transfecting HEK293 cells with the non-silencing (scrambled) shRNAmir vector. **D.** LACE1 KD cells exhibit moderate growth impairment. Stably transfected HEK293 cells were seeded in six-well plates at 5 × 10^4^ cells per well and cultured in DMEM containing 1 μg/ml puromycin. Viable cells were counted every 24 h for a total of 7 d using a Scepter Handheld Automated Cell Counter. **E.** Loss of LACE1 leads to diminished activity of respiratory chain complexes II-IV. The oxygen consumption of digitonin-permeabilized cells was measured at 37°C using an OROBOROS Oxygraph-2k (OROBOROS INSTRUMENTS Corp, Innsbruck, Austria) in 2ml chamber by substrate-inhibitor titration. * *p*<0.05; ** *p*<0.01 **F.** LACE1 KD cells have moderately increased content of complex I and slightly reduced content of complex III. Blue-native PAGE immunoblotting analysis of mitochondria isolated from LACE1 KD cells were performed. Isolated mitochondria were solubilized with 1% (w/v) dodecyl maltoside, resolved on 5-15% blue-native PAGE gels and immunoblotted with antibodies to Ndufb6 (CI), Core 2 (CIII), Cox1 (CIV) and SDHA (CII). CI-CIV denotes four mitochondrial respiratory chain complexes.

The yeast LACE1 homologue (Afg1) was shown to be an inner mitochondrial membrane protein [17]. By performing subcellular fractionation with subsequent immunoblot detection using anti-LACE1 antibody (Abcam), we found that the endogenous processed LACE1 (~50 kDa) is highly enriched in mitochondrial fractions of human embryonic kidney 293 (HEK293) cells (Figure 1A). Immunodetection of mtHSP70 and β-actin was used to control for mitochondrial fractionation efficiency. To find out whether overexpression of wild-type LACE1-FLAG (NM_145315) leads to correct mitochondrial targeting of the fusion protein, we have transiently transfected wild-type HEK293 cells with a C-FLAG-fusion human LACE1 ORF construct (OriGene Technologies). The cells were harvested 48 hours post-transfection and used to prepare mitochondrial fractions. Subjecting mitochondrial fractions to either sonic treatment or alkaline carbonate extraction with subsequent ultracentrifugation and immunoblotting [18] we found that the majority of LACE1-FLAG protein remained in mitochondrial pellet fractions (Figure 1B). CLPP immunoblotting was used as an example of soluble mitochondrial matrix protein, whereas PNPase immunoblotting was used as an example of membrane-associated intermembrane space protein. Taken together, these results indicate that LACE1 is mitochondrial integral membrane protein.

### Knockdown of LACE1 by shRNA leads to reduced PARP cleavage and to diminished activity of mitochondrial respiratory chain

To enable analysis of cellular role of LACE1, we constructed five different HEK293 cell lines stably expressing miR-30-based short hairpin RNAs (shRNAmirs) targeting the human LACE1 transcript (NM_145315). Control cell line was prepared by transfecting HEK293 cells with commercially available scrambled (non-silencing) shRNAmir-containing expression vector RHS436 (Open Biosystems). Western blot analysis with previously validated antibody against LACE1 showed that two of the produced knockdown (KD) cell lines, denoted LACE1sh4 (V3LHS_344953; Open Biosystem) and LACE1sh5 (V3LHS_344954; Open Biosystems), exhibited LACE1 protein levels of less than 10% of control values, respectively (Figure 1C).

Mitochondria play a central role in programmed cell death by regulating the intrinsic apoptotic pathway [19]. We therefore investigated whether loss of LACE1 might have an impact on the rate of programmed cell death. Poly (ADP-ribose) polymerase (PARP) cleavage was assessed as an indicator of ongoing apoptosis [20]. Initially, PARP cleavage under constitutive conditions was assessed. Whole cell lysates were prepared from control and LACE1 KD cells and western blotted with antibody to cleaved PARP. Markedly attenuated levels of cleaved PARP were found in LACE1 KD cells when compared to controls (Figure 1C). This result suggests that LACE1 might be involved in regulation of intrinsic apoptotic pathway.

To assess the effects of LACE1 knockdown on cell viability, we examined the growth rate of LACE1 KD cells over a time course of 7 days. We found only moderate growth impairment associated with the loss of LACE1 (Figure 1D).

Mitochondria are at the center of cellular bioenergetics harboring the energy-converting respiratory chain. Therefore, we next assessed the functional state of mitochondrial respiration in LACE1 KD cells by measuring the oxygen consumption of digitonin-permeabilized cells with the OROBOROS Oxygraph-2k device (OROBOROS INSTRUMENTS Corp, Innsbruck, Austria). We found significantly diminished activity of respiratory chain complex IV in LACE1 KD cells (Figure 1E). However, the activity of complexes II and III was also significantly reduced. In contrast, the activity of respiratory complex I appeared unaffected (Figure 1E). To find out whether the respiratory deficiency was due to diminished amount of selected respiratory complexes in LACE1 KD cells, we performed blue-native PAGE immunoblotting analysis of mitochondria isolated from LACE1 KD cells. Isolated mitochondria were solubilized with 1% (w/v) dodecyl maltoside, resolved on 5-15% blue-native PAGE gels and immunoblotted with antibodies to Cox1, Ndufb6, Core 2 and SDHA. The immunoblots revealed mostly unaffected levels of respiratory chain complexes in LACE1 KD mitochondria, with only moderate increase of complex I and slight reduction of complex III (Figure 1F).

### Loss of LACE1 leads to increased apoptotic resistance whereas its overexpression results in increased apoptotic sensitivity

**Figure 2:**
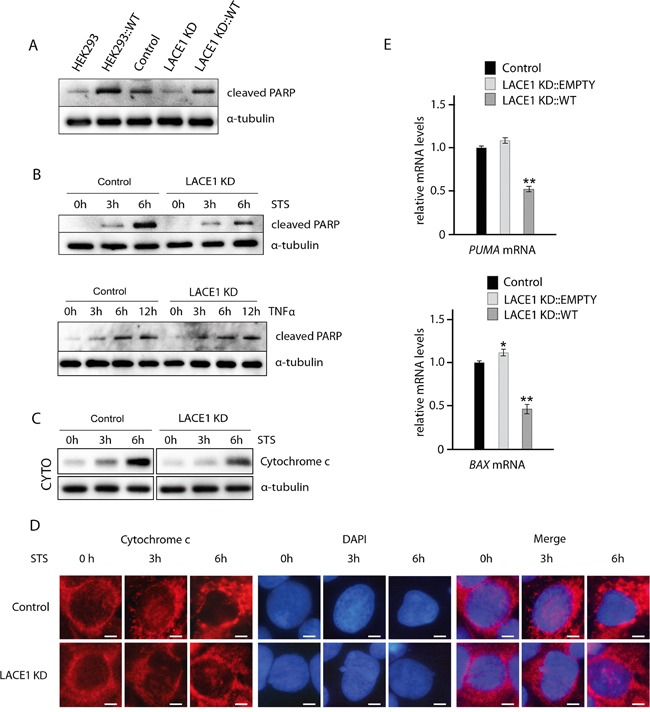
Loss of LACE1 leads to increased apoptotic resistance whereas its overexpression results in increased apoptotic sensitivity **A.** Overexpression of LACE1-FLAG in both wild-type HEK293 cells and LACE1 KD cells results in increased PARP cleavage. Wild-type HEK293 cells and LACE1 KD cells were transfected with LACE1-FLAG expression vector or with the empty vector. Whole-cell lysates were analyzed with western blotting using antibody to cleaved PARP. Antibody to α -tubulin was used to monitor equal protein loading. **B.** LACE1 KD cells exhibit increased resistance to staurosporine-induced apoptosis but respond normally to TNFalpha-treatment. Control and LACE1 KD cells were treated with staurosporine (STS; 2 μM) or TNFalpha (10 ng/ml) and IFN-gamma (80 ng/ml) for 0, 3 and 6 hours. Whole cell lysates were prepared from treated cells and used for immunoblotting with antibody to cleaved PARP. Antibody to α-tubulin was used to control for equal protein loading. **C.** Cells were treated with staurosporine (STS; 2 μM) for 0, 3 and 6 hours and then harvested and used for subcellular fractionation using dounce homogenization and differential centrifugation. The resulting cytoplasmic fractions were subjected to SDS-PAGE western blotting with antibody to cytochrome c. Western blotting of alpha-tubulin was used as loading control. **D.** Cells grown on coverslips were treated with staurosporine (STS; 2 μM) for 0, 3 and 6 hours and then fixed with 4% paraformaldehyde at room temperature and permeabilized with 0.1% Triton X-100. Cells were blocked by 10% Fetal Bovine Serum and primary detection was performed with anti-cytochrome c antibody. After secondary fluorescent detection, cells were analyzed at 24°C using a Nikon Diaphot 200 inverted microscope equipped with a Plan-Apochromat 60×, numerical aperture 0.95, oil objective. The images were acquired with an Olympus DP50 CCD camera and Viewfinder Lite 1.0 software. Bar, 10 μM. **E.** Whereas knockdown of LACE1 leads to moderate upregulation of *PUMA* and *BAX* mRNAs, overexpression of LACE1-FLAG results in their significant downregulation. Relative mRNA quantification was performed using TaqMan Gene Expression Assays according to the manufacturer's instructions (Applied Biosystems). Data were collected in duplicate in two separate runs using a 7300 Real-Time PCR System (Applied Biosystems). HPRT1 (hypoxanthine phosphoribosyltransferase 1), TUBA1A (tubulin, alpha 1a) and TBP (TATA box–binding protein) were used as reference genes.

Since LACE1 KD cells showed substantially diminished constitutive PARP cleavage (Figure 1C), we next assessed staurosporine (STS)-induced apoptosis in these cells. Both control and LACE1 KD cells were treated with 0-h, 3-h and 6-h exposures to staurosporine (2 μM) and used either for preparation of whole cell lysates or for direct immunofluorescence staining. Whole cell lysates were subsequently processed for western blotting with antibody to cleaved PARP (Figure 2B). For immunofluorescence, the cells were stained with antibody to cytochrome c and with DAPI (Figure 2D). Consistently, western blotting of staurosporine treated cells showed reduced levels of cleaved PARP and cytochrome c-immunofluorescence microscopy revealed significantly milder apoptotic changes in STS-treated LACE1 KD cells, compared to identically treated controls (Figure 2B, 2D). To confirm the cytochrome c immunofluorescence results, staurosporine treated cells were subjected to subcellular fractionation and the resulting cytoplasmic fractions were immunoblotted with antibody to cytochrome c. Western blotting detection of alfa-tubulin was used to control for equal cytoplasmic protein loading. The western blots revealed higher cytoplasmic cytochrome c content in control cells, when compared to identically treated LACE1 KD cells, confirming the immunofluorescence results (Figure 2C).

To investigate whether stimulation of the extrinsic apoptotic pathway will affect the control and LACE1 KD cells differently, we used treatment with TNFalpha and IFN-gamma. The ongoing apoptosis was followed by western blotting detection of PARP cleavage. We found similar accumulation of cleaved PARP in both control and LACE1 KD cells after 3, 6 and 12 hours of TNFalpha and IFN-gamma treatment (Figure 2B).

To test whether reintroduction of LACE1 into LACE1 KD background will lead to restoration of apoptotic sensitivity, we have transfected LACE1 KD cells with the LACE1-FLAG expression construct and with the empty expression vector (Figure 2A). The apoptosis was assessed by western blotting detection of cleaved PARP. The blots revealed complete restoration of PARP cleavage in LACE1 KD cells upon introduction of LACE1 but not of the empty expression vector (Figure 2A). To substantiate the apparent pro-apoptotic activity of LACE1, wild-type HEK293 cells were transfected with the LACE1 construct or with the empty vector. Consistently, expression of LACE1 in wild-type HEK293 cells resulted in markedly increased PARP cleavage (Figure 2A). The higher levels of cleaved PARP in wild-type HEK293 cells, when compared to LACE1 KD cells transfected with the identical LACE1-FLAG construct can be attributed to partial RNAi clearance of LACE1-FLAG expression in LACE1 KD background.

Next, we used TaqMan RT-PCR to assess possible changes in *PUMA* and *BAX* transcript levels after knockdown and subsequent reintroduction of LACE1. We found moderately increased *PUMA* and *BAX* mRNA levels in LACE1 KD cells. In contrast, marked reduction in both *PUMA* and *BAX* mRNA levels was found in cells overexpressing LACE1-FLAG (Figure 2E).

### Loss of LACE1 leads to increased PARL and reduced Omi/HTRA2, whereas its overexpression leads to accumulation of p53 in mitochondria and concomitant p53 reduction in nucleus

**Figure 3:**
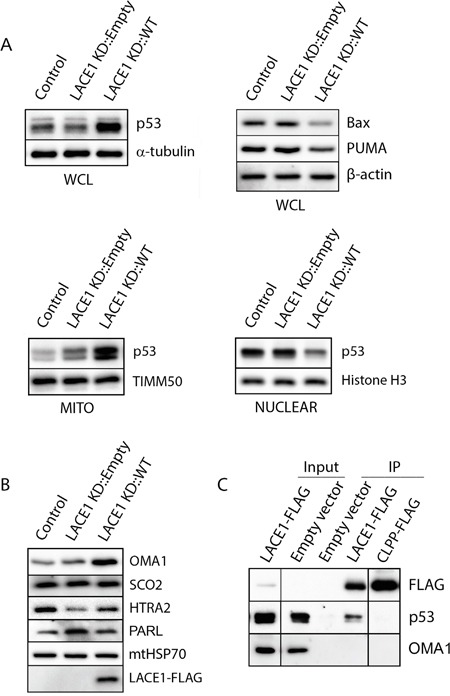
LACE1 expressed in LACE1 KD background physically interacts with p53 and promotes its increased mitochondrial accumulation and nuclear reduction **A.** Overexpression of LACE1 leads to increased mitochondrial accumulation of p53 and its nuclear reduction. Whole-cell lysates (WCL), mitochondrial protein lysates (MITO) and nuclear extracts (NUCLEAR) were prepared from control cell line (Control) and from LACE1 KD cell line transfected with either the empty expression vector or LACE1-FLAG construct and subsequently immunoblotted with antibodies to p53, Bax and PUMA. Equal protein loading and fractionation efficiency was monitored using antibodies to TIMM50, histone H3 and α-tubulin. **B.** Loss of LACE1 leads to upregulation of PARL and downregulation of HTRA2. Mitochondrial protein lysates (MITO) were prepared from control cell line (Control) and from LACE1 KD cell line transfected with either the empty expression vector or LACE1-FLAG construct and subsequently immunoblotted with antibodies to OMA1, SCO2, HTRA2, FLAG, PARL and mtHSP70. **C.** P53 co-immunoprecipitates with LACE1-FLAG expressed in LACE1 KD background. LACE1 KD cells were transiently transfected with the empty expression vector, LACE1-FLAG construct or CLPP-FLAG construct. Mitochondrial fractions were solubilized with 1% Triton X-100 and subjected to anti-FLAG affinity purification. The bound antigens were eluted with 3x FLAG peptide solution under native conditions and processed for western blotting. Five percent (vol) of the purification input was loaded on the gels along with purified samples and immunoblotted with antibodies to FLAG, p53 and CLPP.

Western blotting screen for proteins affected by manipulation of cellular LACE1 levels revealed markedly reduced Omi/HTRA2 and increased PARL in mitochondria of LACE1 KD cells (Figure 3B). Reintroduction of LACE1 ORF into LACE1 KD background led to suppression of the observed protein phenotypes (Figure 3B). Furthermore, overexpression of LACE1-FLAG in LACE1 KD cells led to dramatic mitochondrial accumulation of the p53 tumor suppressor and OMA1 protease and to marked cellular reduction of PUMA and Bax protein levels (Figure 3A). Importantly, the nuclear p53 protein levels were found significantly reduced in LACE1-FLAG overexpressing cells (Figure 3A).

### P53 co-purifies with LACE1-FLAG expressed in LACE1 KD background

To find out which of the observed genetic (functional) interactions of LACE1 found is based on direct protein-protein (physical) interactions we used mitochondria from LACE1 KD cells transfected with the LACE1-FLAG ORF construct to perform affinity co-purification of LACE1-FLAG protein. To control for non-specific results, mitochondrial fraction from LACE1 KD cells transfected with CLPP-FLAG cDNA expression construct (OriGene, Rockville, MD) were used. CLPP (NM_006012) is a proteolytic subunit of the matrix-localized CLPXP complex and thus unlikely to share common interacting partners with LACE1, which is predicted to be an inner membrane protein [17]. Mitochondria were solubilized with 0.5% Triton X-100, incubated with anti-FLAG affinity resin, and co-purifying components were subsequently analyzed using western blotting with battery of validated antibodies against candidate proteins. Highly efficient FLAG protein pull-down was achieved with both LACE1 and CLPP fusion proteins (Figure 3C). Subsequent western blot analysis of co-purifying protein components revealed the p53 tumor suppressor protein to specifically co-purify with LACE1-FLAG, but not the CLPP-FLAG fusion protein. In contrast, the OMA1 protease did not co-purify with any of the two fusion proteins (Figure 3C).

### LACE1 is required for mitomycin c-induced translocation of p53 to mitochondria and its concomitant reduction in nucleus

**Figure 4:**
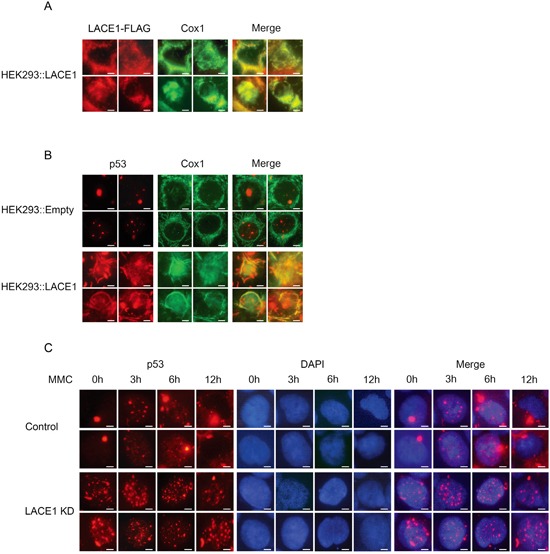
Loss of LACE1 abrogates mitomycin c-induced translocation of p53 into mitochondria whereas its overexpression promotes mitochondrial translocation of p53 **A-B.** Overexpression of LACE1-FLAG in wild-type HEK293 cells promotes increased mitochondrial accumulation of p53. Cells grown on coverslips were transfected with the LACE1-FLAG construct or the empty vector and 48 hours post-transfection were fixed with 4% paraformaldehyde and permeabilized with 0.1% Triton X-100. Cells were then blocked by 10% Fetal Bovine Serum and primary detection was performed with antibodies to FLAG, p53 and COX1. After fluorescent secondary detection, cells were analyzed at 24°C using a Nikon Diaphot 200 inverted microscope equipped with a Plan-Apochromat 60×, numerical aperture 0.95, oil objective. The images were acquired with an Olympus DP50 CCD camera and Viewfinder Lite 1.0 software. Bar, 10 μM. **C.** Loss of LACE1 abrogates mitomycin c-induced mitochondrial translocation of p53. Cells grown on coverslips were treated with mitomycin c (MMC; 5g/mL) for 0, 3, 6 and 12h and then processed for immunofluorescence microscopy essentially as describe in (A-B) except that anti-p53 antibody and DAPI staining were used. Bar, 10 μM.

To confirm the LACE1-induced mitochondrial accumulation of p53, we performed immunofluorescence imaging of wild-type HEK293 cells transfected with the LACE1 ORF construct. Again we found dramatic increase in mitochondrial p53 content in cells with high expression of LACE1-FLAG, as inferred from co-localization of p53 fluorescence signal with that of complex IV subunit COX1 (Figure 4A, 4B). In addition, LACE1-overexpressing HEK293 cells revealed markedly swelled and disrupted mitochondria and leaked mitochondrial content, compared with unaffected mitochondrial reticulum of cells transfected with the empty vector. This is consistent with augmented PARP cleavage after LACE1 overexpression in both wild-type and LACE1 KD HEK293 cells (Figure 2A).

The DNA damage agent mitomycin c (MMC) can induce mitochondrial accumulation of p53 protein [21]. As our results have indicated that LACE1 may be required for mitochondrial translocation and/or accumulation of p53, we used MMC treatment to find out whether mitochondrial translocation of p53 is compromised in LACE1 KD cells. Both control and LACE1 KD cells were treated with MMC (5g/mL) for 0, 3, 6 and 12h and then analyzed with anti-p53 and DAPI immunofluorescence microscopy. The resulting images revealed marked cytoplasmic (mitochondrial) accumulation of p53 in control cells after 6- and 12-h exposures to MMC. This increase in cytoplasmic p53 protein was accompanied by marked reduction in nuclear p53 protein content (Figure 4C). In contrast, neither the increase in cytoplasmic p53 nor the concomitant reduction in nuclear p53 content could be seen in identically treated LACE1 KD cells (Figure 4C). Additionally, the immunofluorescence images of untreated (0h) cells revealed markedly altered nuclear distribution pattern of p53 protein in LACE1 KD cells, compared to untreated (0h) control cells. In contrast to highly localized nuclear distribution of p53 protein in control cells, the nuclear p53 signal was significantly more scattered in LACE1 KD cells, with multiple dots representing the p53 protein signal (Figure 4C). Similar redistribution of p53 protein signal could be seen in control cells only after 3 and 6h treatment with MMC.

### The LACE1-mediated apoptosis is dependent on p53

**Figure 5:**
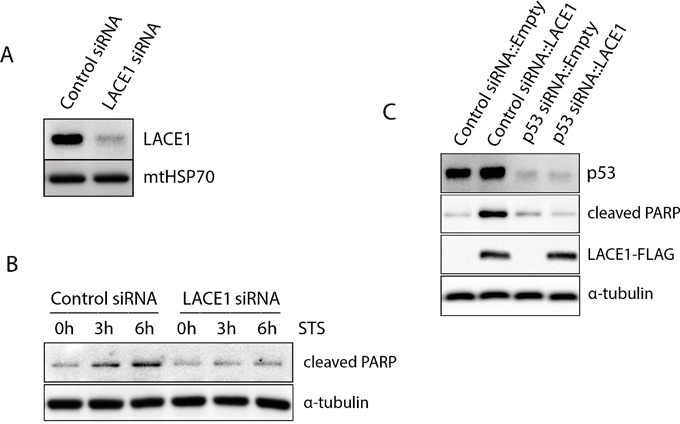
The LACE1-mediated apoptosis is dependent on p53 **A.** Transfection of human dermal fibroblasts with LACE1 stealth siRNA leads to efficient knockdown of LACE1 protein. Normal human dermal fibroblasts (NHDFs) were transfected with control and LACE1 siRNA, harvested 48 hours post-transfection and processed for western blotting. The blots were developed with antibodies to LACE1 and mtHSP70. **B.** Knockdown of LACE1 in NHDFs by siRNA renders cells resistant towards STS-induced apoptosis. Both control siRNA- and LACE1 siRNA-transfected NHDFs were treated with 0-h, 3-h and 6-h exposures to staurosporine (2 μM). Whole-cell lysates were subsequently western blotted with antibody to cleaved PARP. Equal protein loading was controlled with antibody to α-tubulin. **C.** The LACE1-mediated apoptosis is dependent on p53. NHDFs in which p53 was downregulated using siRNA as well as appropriate siRNA control NHDFs were transfected with either LACE1-FLAG or empty vector constructs. The resulting whole-cell extracts were western blotted with antibodies to p53, cleaved PARP, FLAG. Equal protein loading was monitored with α-tubulin detection.

To find out whether the pro-apoptotic activity of LACE1 requires p53 and to verify the previous results using cells with normal p53 signaling, we used Stealth™ siRNA knockdown in normal human dermal fibroblasts (NHDFs). First, the NHDFs were transfected with LACE1 siRNA and the staurosporine (STS)-induced apoptosis was assessed. Both control siRNA and LACE1 siRNA NHDFs were treated with 0-h, 3-h and 6-h exposures to staurosporine (2 μM). Whole-cell lysates were subsequently processed for western blotting with antibody to cleaved PARP. Consistent with the results on HEK293 cells, siRNA-mediated knockdown of LACE1 in NHDFs resulted in significantly increased resistance of the cells to STS-induced apoptosis (Figure 5A, 5B). Next, we generated p53 siRNA knockdown NHDFs as well as appropriate siRNA control cells. Again, substantial p53 protein downregulation was obtained using stealth siRNA expression (Figure 5C). Both p53 knockdown cells and the appropriate siRNA control cells were subsequently transfected with either LACE1-FLAG or empty vector constructs. The resulting whole-cell extracts were western blotted with antibodies to p53, cleaved PARP, FLAG and α-tubulin. Consistent with previous results, the western blots showed markedly increased amount of cleaved PARP in control siRNA cells overexpressing LACE1-FLAG (Figure 5C). Importantly, the LACE1-FLAG overexpression failed to induce PARP cleavage in p53 knockdown cells (Figure 5C).

## DISCUSSION

p53 is a major cellular tumor suppressor protein that besides its well characterized nuclear-based transcription-dependent function has the ability to translocate to mitochondria and trigger here cell death via transcription-independent manner. In the present study, we have shown an important role in subcellular partitioning of p53 and transcription-independent p53 apoptotic signaling for LACE1 protein, a putative mitochondrial ATPase. We show here that LACE1 exhibits significant pro-apoptotic activity in human HEK293 cells and normal human dermal fibroblasts and that it is required for normal function of the mitochondrial respiratory chain. We further demonstrate that LACE1 physically interacts with p53 and is required for mitochondrial translocation of p53 protein. We show that the LACE1-induced mitochondrial accumulation of p53 is associated with concomitant reduction in p53 nuclear content and activity. Using siRNA knockdown of p53 in normal human dermal fibroblasts we show that the LACE1-mediated apoptosis is dependent on p53.

Amongst the most obvious features of LACE1 knockdown cells is the increased resistance to apoptosis and diminished activity of the mitochondrial respiratory chain. Since overexpression of LACE1 in both LACE1 knockdown cells as well as wild-type HEK293 cells led not only to suppression of the apoptotic defect but to major apoptotic phenotype point to significant pro-apoptotic character of human LACE1. Importantly, the LACE1-mediated apoptosis is shown to depend on p53 in normal human dermal fibroblasts. Furthermore, although increasingly resistant to intrinsic apoptosis, the LACE1 KD cells are fully susceptible to TNFalpha-induced cell death. The mitochondrial rhomboid protease PARL functions anti-apoptotic in mammalian cells by controlling cristae remodeling and cytochrome c release [22]. Hence, the markedly increased levels of PARL in LACE1 knockdown cells are consistent with their increased apoptotic resistance. Conversely, the intermembrane space serine protease Omi/HTRA2 shows significant pro-apoptotic activity, mainly through degradation of IAP (inhibitors of apoptosis) proteins [23]. Thus, the downregulation of Omi/HTRA2 along with concomitant upregulation of PARL is also consistent with the increased apoptotic resistance of LACE1 deficient cells.

The tumor suppressor protein p53 is activated upon various stress stimuli to either support cell survival or promote apoptosis. Besides its well established nuclear, transcription-dependent function, p53 was shown to act also extranuclearly. Indeed, in response to acute cellular stress or even acute exercise, p53 can rapidly translocate into mitochondria and activate here apoptosis via fast, transcription-independent manner [24, 25]. Several lines of experimental evidence support the direct involvement of LACE1 in mitochondrial partition of p53 protein. First, overexpression of LACE1 leads to dramatic increase in mitochondria-associated p53 and to concomitant reduction in nuclear p53 content accompanied by reduced expression of p53 transcription target genes BAX and PUMA. Second, the DNA damage agent mitomycin c fails to induce mitochondrial translocation and concomitant nuclear reduction of p53 in LACE1 knockdown cells. Finally, LACE1-FLAG protein expressed in LACE1 knockdown cells physically interacts with p53. The role of LACE1 in mitochondrial partition of p53 is consistent with both the significant pro-apoptotic character of LACE1 protein and with the previously reported tumor-suppressor properties of LACE1 gene region [15]. The unaffected levels of SCO2 in LACE1 overexpressing cells further confirm the extranuclear, transcription-independent character of LACE1-induced p53 upregulation. Besides its function in complex IV assembly, the SCO2 metallochaperone was identified as a p53 transcription target gene required for shift of cellular metabolism from glycolysis to increased OXPHOS utilization [26–28]. On the other hand, positive genetic interaction between the mitochondrial metallopeptidase OMA1 and p53 was observed in OVCA cells, which is consistent with LACE1-induced OMA1 upregulation seen in our HEK293 knockdown model [29]. Besides its specific role in mitochondrial homeostasis and apoptosis, the subcellular partitioning of p53 into mitochondria has the potential to substantially affect its nuclear activity [30]. Indeed, the pattern of nuclear distribution of p53 protein is substantially affected in LACE1 KD cells. On the other hand, expression of p53 transcription target genes BAX and PUMA was found significantly diminished in LACE1 overexpressing cells. Several mechanisms have been proposed to drive the mitochondrial translocation of p53 [24, 25, 30]. Similarly to our report, one of them utilizing the mitochondrial disulfide relay protein CHCHD4 was shown to be connected to mitochondrial respiratory capacity [30]. The yeast homologue of human LACE1 (Afg1) was shown to be required for normal activity of respiratory complexes III and IV [17]. Similar to the yeast Afg1 deletion strain, the rather moderate respiratory defect of human LACE1 knockdown cells suggests possible redundant character of LACE1 role in respiratory chain maintenance. Given that ATP- or ADP-binding state may change biochemical property of LACE1, it is interesting to speculate that LACE1-p53 interaction could serve as an immediate apoptotic response to altered mitochondrial ATP levels. Further work is required to address the effects of LACE1-mediated subcellular p53 partitioning towards cell metabolism and proliferation.

## MATERIALS AND METHODS

### Cell culture and transfection

Human embryonic kidney cells (HEK293) and normal human dermal fibroblasts (NHDFs) were obtained from the American Type Culture Collection (Rockville, MD) and maintained in high-glucose DMEM (PAA Laboratories, Pasching, Austria) supplemented with 10% (vol/vol) fetal bovine serum Gold (PAA Laboratories) at 37°C in a 5% (vol/vol) CO2 atmosphere.

### shRNA, siRNA and gene expression constructs

A negative control (scrambled) pGIPZ shRNAmir construct and five different pGIPZ shRNAmir constructs targeting the human LACE1 transcript (NM_145315) were obtained from Open Biosystems (GE Dharmacon). To generate stable LACE1 KD cells, subconfluent HEK293 cells (10^7^) were transfected by electroporation using Nucleofector™ (Lonza, Walkersville, MD) with cell specific kit according to manufacturer's instructions, and stably expressing cells were selected using puromycin at a concentration of 1.5 μg/ml over a period of 3 wk. Western blot analysis was used to evaluate the efficiency of LACE1 knockdown at the protein level in each of the stable cell lines (monoclonal anti-FLAG M2 antibody, Sigma).

A negative control (scrambled) Stealth RNAi siRNA and functionally validated Stealth RNAi siRNA duplexes targeting the human LACE1 and p53 transcripts were purchased from Invitrogen. The RNAiMAX Transfection reagent (Invitrogen) was used for transient transfection of cells. The transfected cells were harvested 48 hours post-transfection and either stored at −80°C or used for subsequent analyses.

The C-fusion Myc-FLAG-tagged open reading frame (ORF) expression constructs pCMV6-LACE1 containing the human LACE1 cDNA (NM_145315) ORF sequence and the pCMV6-CLPP containing the human CLPP cDNA ORF sequence ((NM_006012) were purchased from OriGene (Rockville, MD). The fidelity of all constructs was confirmed by automated DNA sequencing. Cells (10^7^) were transiently transfected with the gene expression constructs using the Lipofectamine® 2000 Transfection Reagent (Life Technologies, USA) according to manufacturer's instructions. Transfected cells were harvested 48-h post-transfection and either directly processed for analyses or stored at −80°C.

### Fluorescence microscopy

Living HEK293 cells grown on coverslips (Willco Wells, Netherlands) were fixed with 4% paraformaldehyde (Affymetrix; 19943) in phosphate buffered saline (PBS; Lonza; BE17-517Q) at room temperature (RT) for 20 min and permeabilized with 0.1% Triton X-100 (Sigma Aldrich; X100) in PBS at RT for 15 min, followed by rinse in PBS. Cells were blocked by 10% HyClone Fetal Bovine Serum (GE Healthcare Life Sciences; SV30160) in PBS at RT for 60 min. Primary detection was performed with anti-COX1 (Abcam; Ab14705; 1:250), anti-cytochrome c (Abcam; Ab110325; 1:200) or anti-p53 (Cell Signaling; 2527S; 1:1600) primary antibodies. Cells were incubated with primary antibody in 10% HyClone Fetal Bovine Serum in PBS at 4°C over night, followed by rinse in PBS. Secondary detection were performed with Alexa Fluor® 488 IgG2α (Life Technologies; A21131; 1:500), Alexa Fluor® 594 IgG2α (Life Technologies; A21135; 1:500) or Alexa Fluor® 594 IgG (H+L) (Cell Signaling; 8889; 1:500). Cells were incubated with secondary antibody in 10% HyClone Fetal Bovine Serum in PBS at RT for 2h covered with cellophane, followed by rinse in PBS. Cells were analyzed at 24°C using a Nikon Diaphot 200 inverted microscope (Nikon, Tokyo, Japan) equipped with a Plan-Apochromat 60×, numerical aperture 0.95, oil objective (Carl Zeiss, Wetzlar, Germany). The images were acquired with an Olympus DP50 CCD camera (Olympus, Milan, Italy) and Viewfinder Lite 1.0 software (Pixera, Santa Clara, CA). For fluorescence staining, living, intact HEK293 cells were incubated with 10 nM MitoTracker Red CMX Ros (Life Technologies; M-7512) or DAPI (Life Technologies; D1306) for 15 min in phosphate-buffered saline (PBS) and analyzed at 24°C using identical equipment as above.

### Cell death analysis

Transfected HEK293 cells or human skin fibroblasts were treated with exposure to staurosporine (STS) (2 μM) or TNFalpha (10 ng/ml) and IFN-gamma (80 ng/ml) for 0, 3, and 6 h and whole cell lysates were analyzed by immunoblotting with antibody to cleaved PARP.

### The assessment of cell proliferation

Stably transfected HEK293 cells were seeded in six-well plates at 5 × 10^4^ cells per well and cultured in DMEM containing 1 μg/ml puromycin. The medium was changed on the second, fourth, and sixth days. Viable cells were counted every 24 h for a total of 7 d using a Scepter Handheld Automated Cell Counter (Millipore, Billerica, MA).

### Reverse transcription and quantitative RT-PCR

Total RNA isolation from HEK293 cells and first-strand cDNA synthesis was performed essentially as described before [31]. Relative mRNA quantification was performed using TaqMan Gene Expression Assays according to the manufacturer's instructions (Applied Biosystems). Data were collected in duplicate in two separate runs using a 7300 Real-Time PCR System (Applied Biosystems). HPRT1 (hypoxanthine phosphoribosyltransferase 1), TUBA1A (tubulin, alpha 1a) and TBP (TATA box–binding protein) were used as reference genes.

### Co-immunoprecipitation experiments

For anti-FLAG co-immunoprecipitation, the LACE1 KD cells were transiently transfected with the LACE1-FLAG construct, with the CLPP-FLAG construct or with the empty parental pCMV6 vector. Equivalent transfection efficiency was monitored by Western blotting with the anti–FLAG M2 monoclonal antibody (Sigma-Aldrich, USA). The transfected cells were harvested at 48-h post-transfection and then used to prepare mitochondrial fractions. Isolated mitochondria were solubilized using 1% Triton X-100 in TBS containing 1% protease inhibitor cocktail at a protein concentration of 2 mg/ml. The resulting cleared extracts were incubated for 2-h with washed anti–FLAG M2 affinity beads (Sigma-Aldrich, USA) at 4°C with gentle agitation. The affinity beads containing the bound antigens were then washed five times with TBS containing 0.1% Triton X-100 and the bound material was eluted under non-denaturing conditions with 3× FLAG peptide solution and routinely processed for SDS–PAGE Western blotting.

### Electrophoresis and Western blotting

Electrophoresis and Western blotting were performed essentially as described previously [31, 32], except that a semi-dry blotter FastBlot B64 (Biometra, Germany) was used for electrotransfer. Membranes were blocked with 0.2% I-block solution (Applied Biosystems, USA) to prevent nonspecific interactions. Blots were subsequently developed using either West Femto or West Pico chemiluminescent substrates (Pierce, Rockford, IL). Western blot signals were acquired using the G:BOX imaging systeme (Syngene, UK). The resulting digital images were analyzed and quantified using the Quantity One application (Bio-Rad).

### Antibodies

The monoclonal antibody against human LACE1 was obtained from Abcam, UK. The M2 monoclonal antibody to FLAG epitope was obtained from Sigma Aldrich, USA. Antibody to mtHSP70 was from Lonza, Switzerland and antibodies to p53, Cl-PARP and α-tubulin were from Cell Signaling Technology, USA. Antibodies to OMA1, TIMM50, Omi/HTRA2, PARL, CLPP and PNPase were from Abcam, UK. Antibody to SCO2 was from previous studies [33, 34].

### Mitochondrial isolation and subfractionation

Mitochondrial fractions were isolated from HEK293 cells using cell disruption by hypo-osmotic swelling coupled to Dounce homogenization, removal of nuclear contamination by low-speed centrifugation (2,000 × *g*) and fractionation in discontinuous sucrose density gradient (1-1.5 M) by ultracentrifugation (85,000 × *g*) essentially as described herein [35]. Optimal cell disruption was monitored using microscopy and nuclear contamination was monitored by Western blotting. To prepare submitochondrial fractions, mitochondria at a protein concentration of 1 mg/ml were either sonicated (Ultrasonic Homogenizer 4710 Series; Cole-Parmer, Chicago, IL) or treated with 100 mM sodium carbonate (pH 11.5) and were then centrifuged for 1 h at 144,000 × *g*. The resulting supernatant fractions were trichloroacetic acid (TCA) precipitated and subsequently dissolved in SDS–PAGE sample buffer along with the washed pellet fractions.

### Preparation of nuclear extracts

The cells were washed with PBS, pelleted by centrifugation and resuspended in the cell lysis buffer [10 mM HEPES (pH 7.5), 10 mM KCl, 0.1 mM EDTA, 1 mM dithiothreitol (DTT), 0.5% Nonidet-40 and 0.5 mM PMSF] along with the protease inhibitor cocktail (Sigma) and allowed to swell on ice for 15-20 min. The cells were then vortexed to disrupt cell membranes and then centrifuged at 12,000 × g at 4°C for 10 min. The pelleted nuclei were washed with the cell lysis buffer and resuspended in the nuclear extraction buffer [20 mM HEPES (pH 7.5), 400 mM NaCl, 1 mM EDTA, 1 mM DTT, 1 mM PMSF] with protease inhibitor cocktail and incubated in ice for 30 min. The nuclear extract was collected by centrifugation at 12,000 × g for 15 min at 4°C.

### High-resolution respirometry

The oxygen consumption of digitonin-permeabilized cells was measured at 37°C using an OROBOROS Oxygraph-2k (OROBOROS INSTRUMENTS Corp, Innsbruck, Austria) in 2ml chamber with KCl-based medium containing 80mM KCl, 10mM Tris, 3mM MgCl2, 1mM EDTA, 5mM potassium phosphate (pH 7.4). The measurements were carried out after permeabilization of cells (manual titration) by approx. 4.1 μM digitonin, protein load per chamber ranging between 0.5 – 0.9 mg, with final concentrations of substrates and inhibitors as follows: 10mM glutamate, 2.5mM malate (CI-ADP), 1mM ADP (CI/+ADP), 10mM succinate (CII), 4mM ascorbate and 0.4mM TMPD (N,N,N′,N′-tetramethyl-1,4-phenylenediamine) (CIV), 0.5μM rotenone, 1μM antimycine A. To control for intactness of mitochondrial membranes, cytochrome *c* (2.5μM) was added after succinate. Respiration was uncoupled by 200 nM FCCP titration steps, maximum 1.5 μM (CIVu), and finally inhibited by the addition of sodium azide (10 mM).

### Statistical analysis

For analysis of significance (high-resolution respirometry), Cox's F-test was applied, which is a non-parametric test suitable for lower number of cases with non-normal data distribution (either exponential or Weilbull). *p*<0.05 was considered as statistically significant, null hypothesis was rejected and significance is denoted as follows: * *p<0.05*; ** *p<0.01*.
